# Impact of secondary salts, temperature, and pH on the colloidal stability of graphene oxide in water†

**DOI:** 10.1039/d2na00070a

**Published:** 2022-04-22

**Authors:** Sergio Mancillas-Salas, Ana C. Reynosa-Martinez, J. Barroso-Flores, Eddie Lopez-Honorato

**Affiliations:** Centro de Investigación y de Estudios Avanzados del IPN (CINVESTAV) Unidad Saltillo, AV. Industria Metalúrgica 1062 Ramos Arizpe 25900 Mexico; Instituto de Química, Universidad Nacional Autónoma de México Circuito Exterior Ciudad Universitaria, México 04510 D.F. Mexico; Centro Conjunto de Investigación en Química Sustentable UAEM-UNAM, Carretera Toluca-Atlacomulco Km 14.5, Unidad San Cayetano Toluca Estado de México 50200 Mexico; Oak Ridge National Laboratory Oak Ridge TN 37831 USA honoratole@ornl.gov

## Abstract

The stability of graphene oxide (GO) in water is extremely relevant because of its application as an adsorbent material, as well as for its fate and behavior in the environment. Zeta potential was used to study the effect of secondary salts (carbonate, sulfate, and phosphate), temperature (20 to 60 °C), and pH (5 to 9) on the stability of six different GOs produced from natural, synthetic, and amorphous graphite—with and without the use of attrition milling. Generally, GOs produced with attrition-milled graphites had lower ζ-potentials than their unmilled counterparts because of their smaller particle sizes and higher concentration of oxygen-containing functional groups. It was observed that GO produced from graphite and synthetic graphite had ζ-potential values lower than −30 mV, even at 30 °C. However, it was observed that all the GOs studied were unstable in the presence of carbonate and sulfate salts at concentrations between 170 and 1695 mg L^−1^, as they reached a ζ-potential of −4.1 mV. Density-functional theory electronic structure calculations suggested that the instability of GO in the presence of carbonate and sulfate was caused by the abstraction of a proton resulting in interaction energies *E*_int_ of 28.3 and 168.9 kJ mol^−1^, respectively. Our results suggest that temperatures above 30 °C, as well as carbonate and sulfate salts at concentrations relevant to arid and semi-arid regions, could promote the formation of agglomerates of GO, thus limiting its use and mobility in water.

## Abstract 2

Water conditions in arid and semi-arid regions would agglomerate graphene oxide regardless of the particle size and oxidation degree.

## Graphical abstract

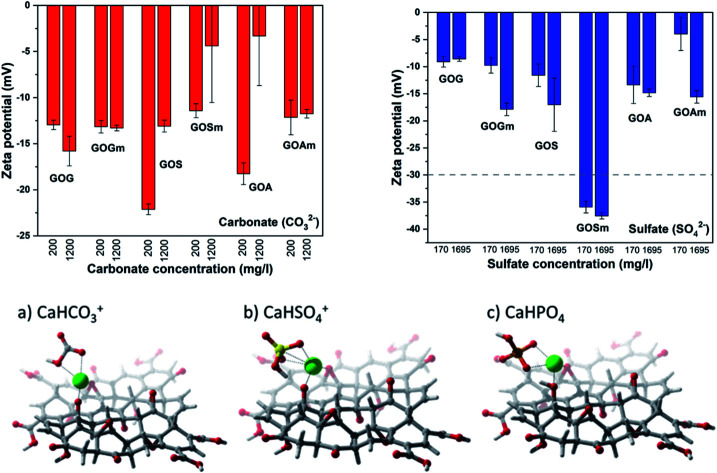

## Introduction

Graphene oxide (GO) is a 2D carbon nanomaterial with sp^2^ and sp^3^ hybridization, functionalized with hydroxyl, carbonyl, and epoxy functional groups.^1^ The presence of these functional groups confers a hydrophilic behavior to this nanomaterial and the possibility to form stable suspensions in water.^1,2^ These functional groups also facilitate GO's use as an adsorbent material for the separation of radionuclides in nuclear waste or other contaminants in water, for the production of membranes for ion and molecular separation, and for nanofluids to improve thermal transport in solar thermal energy.^3–6^ Furthermore, the stability of GO in aqueous media is also relevant from an environmental perspective, as it affects its transport and fate.^7–11^

The amphiphilic nature of GO can be tuned by modifying the C/O ratio, for example, by varying the concentration of the oxidizing agent during its synthesis,^2,3,6,12^ which has resulted in the use of GO with a wide range of oxidation degrees and microstructures.^3,13–15^ Additionally, the stability of GO in water is affected by water chemistry, for example, by ions occupying the active sites of GO by modifying its surface charge density.^16,17^ This results in the reduction of the repulsion force and the intensification of the attractive force, which promotes the aggregation of GO sheets and their subsequent precipitation.^18^ Previous studies on the stability, aggregation, and transport of GO in water focused on the study of the ionic strength of salts such as sodium (NaCl), manganese (MgCl_2_), and calcium chloride (CaCl_2_), at concentrations of 20, 50, and 100 mM for NaCl; 0.3 to 30 mM for MgCl_2_; and 0.1 to 10 mg L^−1^ for CaCl_2_.^18–23^ However, it is also necessary to study the effect of other secondary salts such as carbonate, sulfate, and phosphate on the stability of GO in water—particularly at concentrations relevant to environments such as arid and semi-arid regions, where the concentrations of these salts can be as high as 1293 mg L^−1^, 1695 mg L^−1^, and 30 mg L^−1^ for carbonate, sulfate, and phosphate, respectively.^24,25^ Furthermore, considering that water surface temperatures can reach values close to 70 °C in coastal semi-arid areas and 40.7 °C in arid regions,^24–26^ temperature could also have an important impact on GO stability in water. Therefore, it is necessary to understand how different types of GO interact with secondary salts, as well as the effect of pH and temperature on the stability of GO in water, since these variables could have important implications for its applications, transport, and fate in the environment.

In this work, six GOs were synthesized from different graphitic sources to establish a relationship between the characteristics of GO and its stability in water. Graphite flakes (GFs), synthetic graphite (SG), and amorphous graphite (AG) were used as starting materials in the synthesis of GO. These graphites were also modified by attrition milling to increase their degree of oxidation, without the use of a higher amount of oxidizing agents.^3^ The stability of these GO-nanofluid suspensions was evaluated using zeta potential, hereinafter referred to as ζ-potential. It was observed that all the GOs from milled graphites were more stable in aqueous media (−35 to −43.1 mV) regardless of the pH tested (5, 7, and 9), as they maintained their suspension stability even when the temperature rose to 50 °C. However, even though GOs from milled graphite showed a higher degree of oxidation, their stability was negatively affected by the high concentration of secondary salts such as carbonate (1200 mg L^−1^) and sulfate (1695 mg L^−1^): their ζ-potential increased to −4.5 and −15.7 mV, respectively. Conversely, only in the presence of a low concentration of phosphate (30 mg L^−1^) did the nanofluids keep their stability, with a ζ-potential of −35.9 mV. Additionally, density-functional theory (DFT) calculations were performed to understand the interaction between GO and these secondary salts in water.

## Experimental section

### Graphene oxide synthesis

Graphene oxide was synthesized following an improved Hummers' method proposed by Marcano,^27^ using three types of graphite as starting materials: GF (Sigma Aldrich 95%), SG, and AG (both from Carbograf Industrial). In a separate test, these three graphite materials were first attrition milled for 24 hours in a built-in-house attrition mill (245 cm^3^ vial capacity covered with Teflon) using 10 wt% methanol as solvent, 0.3 mm YSZ milling media, and 450 rpm at 5 °C, using a water recirculating chiller (IKA RC 5 basic).^28^

The GO synthesis was performed as follows. First, H_2_SO_4_ (95–98% Sigma Aldrich) and H_3_PO_4_ (85% J. T. Baker) were mixed in a 9 : 1 ratio with 3 g of graphite. After 30 minutes of stirring, KMnO_4_ (99% Sigma Aldrich) was added while maintaining the temperature at 50 °C for 24 h. Subsequently, the mixture was cooled to 2 °C, and 3 mL of H_2_O_2_ at 30% (Jalmek) were added. The mixture was then diluted with deionized water to reach a pH of 1. The solid material was washed twice with HCl (36.5–38%, Jalmek) at 30% v/v, and then with deionized water, and finally with ethanol (99.5% Jalmek). The material was flocculated using diethyl ether (99%, Jalmek) and centrifuged at 3500 rpm for 30 minutes (XC-2450 PREMIERE). Afterward, the solid material was dispersed in ethanol and exfoliated in an ultrasonic bath (Branson 3800, Frequency 40 kHz/Sonic Power 110) for 1 h and dried overnight at 80 °C. The material was ground (agate mortar) and sieved (100 mesh) before characterization.

### Graphene oxide characterization

GO was characterized by X-ray diffraction (XRD) (Phillips X'Pert, Cu Kα *λ* = 1.5418 Å, 40 kV, and 30 mA), Fourier transform infrared spectroscopy (FTIR) (PerkinElmer Frontier/NIR), and X-ray photoelectron spectroscopy (XPS) (Thermo Scientific K-Alpha) with a 1.9 × 10^−7^ mbar vacuum chamber and an aluminum anode as the X-ray monochromatic source with a radiation energy of 1486.68 eV. It was calibrated with the binding energy of carbon, 284 eV. XPS data in the C1s region were analyzed using the CasaXPS program. A Shirley-type baseline and Gaussian and 30% Lorentzian functions were used to fit the bands for sp2 and sp3 bonds (284.6 eV), C–OH (285.7 eV), C–O–C (286.7 eV), C=O (287.6 eV), and COOH (289.0 eV).^29–31^ The microstructure was characterized by scanning electron microscopy (SEM) using a JEOL JSM-7800F Prime and by transmission electron microscopy (TEM) using an FEI TALOS at 200 kV.

ζ-Potential (Malvern Panalytical Zetasizer) was measured employing a 0.02 wt% concentration at pH 5, 7, and 9, at temperatures of 20, 30, 40, 50, and 60 °C. Calcium carbonate CaCO_3_ (99% Sigma Aldrich) solutions were prepared at concentrations of 200 and 1200 mg L^−1^, whereas for sulfate (SO_4_^2−^), two concentrations of CaSO_4_·2H_2_O (98% Sigma Aldrich) were prepared—170 and 1695 mg L^−1^. Finally, two phosphate solutions (PO_4_^3−^) of 1.5 and 30 mg L^−1^ of Ca_3_(PO_4_)_2_ (96% Sigma Aldrich) were prepared. These salt concentrations were chosen to be representative of semi-desertic areas from an analysis of common salts contained in the water of the Chihuahuan desert in the state of Coahuila at the northeast of Mexico.^24,25^

### Computational methods

DFT calculations were performed using the Gaussian 09 Revision E.01 suite of programs.^32^ Structures were built from the literature,^33^ and the calcium salts were manually docked on top. Each structure was optimized at the LC-ωPBE/6-31G(d,p) level of theory, and the interaction energy, *E*_int_, between secondary salts and GO was calculated with the Natural Bond Orbital (NBO) deletion scheme as included in the NBO3.1 program provided with the aforementioned suite.^34^

## Results and discussion

**Fig. 1:**
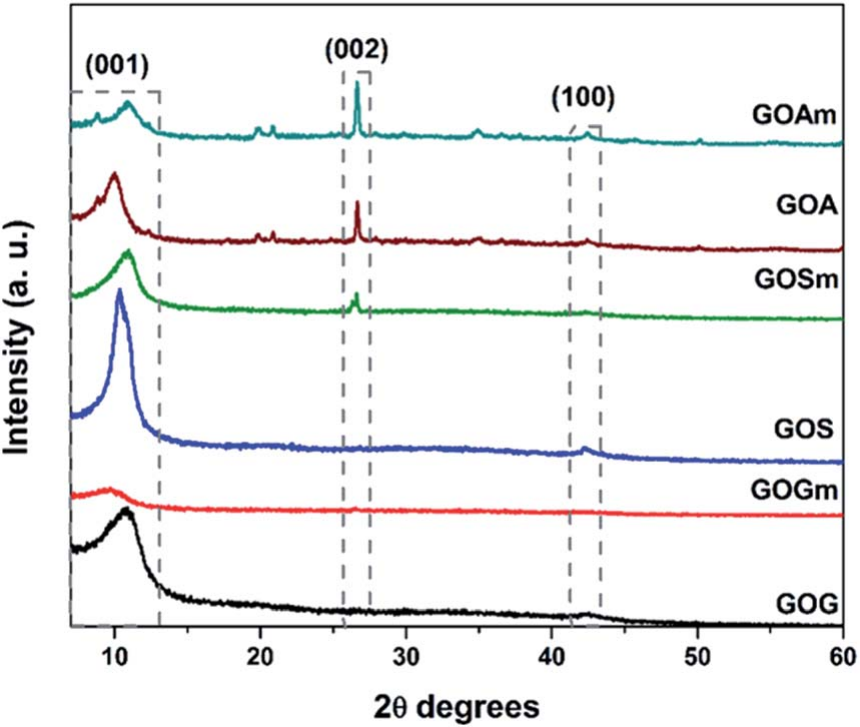
XRD patterns of GO synthesized from graphite flakes (GOG), milled graphite flakes (GOGm), synthetic graphite (GOS), milled synthetic graphite (GOSm), amorphous graphite (GOA), and milled amorphous graphite (GOAm).

Fig. 1 shows the XRD patterns of GO synthesized from graphite flakes (GOG), milled graphite flakes (GOGm), synthetic graphite (GOS), milled synthetic graphite (GOSm), amorphous graphite (GOA), and milled amorphous graphite (GOAm). The presence of the (001) reflection at around 10° observed for all the samples is a feature generally used to confirm the formation of GO.^35^ The (002) reflection at 26.6° corresponds to the ordered structure of graphite whose presence indicates remnants of the graphite structure from the original material, which suggests that the oxidation process did not occur homogeneously.^35,36^ Additionally, the (100) reflection at 42.5° is associated with the turbostratic or disordered graphite, with disorder in the domain parallel to the basal plane.^36^ This last reflection is observed only in GOs synthesized from SG and AG (pristine and milled) since these two materials have a higher degree of disorder than graphite flakes.

Fig. 1S (ESI†) shows the FTIR spectra of all GOs synthesized. In these spectra, it was possible to identify the bands corresponding to the C–H vibration at 875 cm^−1^, C–O stretching at 1100 cm^−1^, C=C at 1600 cm^−1^, C=O from carbonyls at 1750 cm^−1^, and –OH at approximately 3300 cm^−1^; all these bands are characteristic of GO.^37^ Additionally, GOS and GOSm showed a peak at around 1225 cm^−1^, corresponding to the stretching mode of epoxy groups (C–O–C) located on the basal planes of the graphene sheets.^38^

**Fig. 2:**
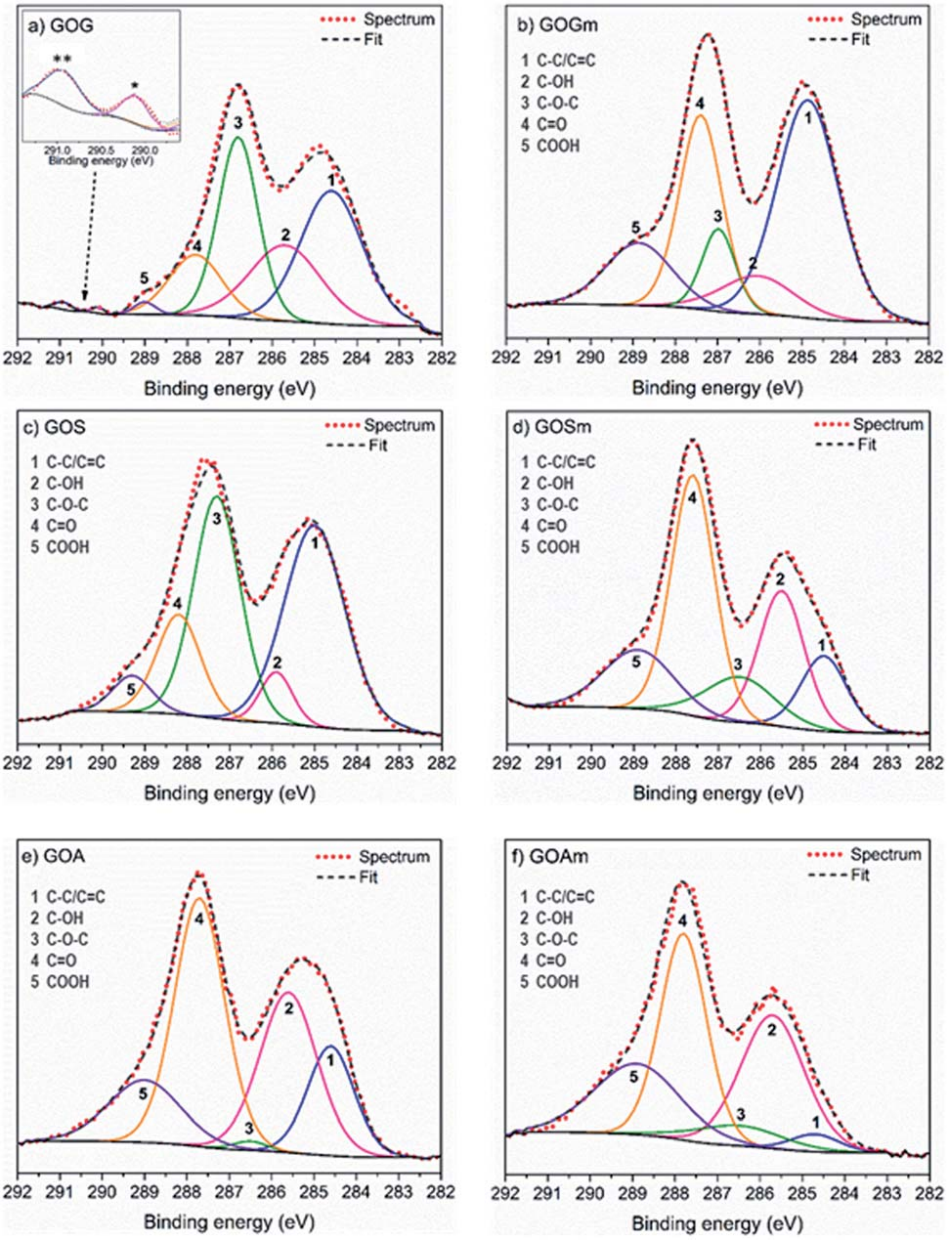
XPS spectra fit of GO synthesized from (a) graphite flakes (GOG), (b) milled graphite flakes (GOGm), (c) synthetic graphite (GOS), (d) milled synthetic graphite (GOSm), (e) amorphous graphite (GOA), and (f) milled amorphous graphite (GOAm).

A more detailed analysis of the functional groups in GO was achieved using XPS, as shown in Fig. 2 and Table 1. It was observed that GOGm, in comparison with GOG, had a higher concentration of C=O and COOH functional groups: they increased from 13 and 1.1% to 26.3 and 13.2%, respectively. Conversely, the content of –OH and C–O–C decreased from 22.1 and 29.2% to 8.9 and 8.7%, respectively. This difference in the concentration of functional groups could be related to the progress of the oxidation of graphene oxide, since the C–OH group is the first group formed during the oxidation process. However, as the oxidation continues COOH, C=O and C–O–C groups are generated.^38^ In addition to the bands corresponding to the oxygenated functional groups, the presence of two bands in GOG (marked with asterisks in Fig. 2) between 290 and 291 eV was also observed. These bands are commonly attributed to shake-up satellites of aromatic carbons, probably from the graphene structure that was not oxidized.^39^ On the other hand, the GO from milled synthetic graphite (GOSm) had a higher concentration of oxygenated functional groups, 22.6% C–OH, 11.6% C–O–C, 38% C=O, and 15.2% COOH, compared to the GO produced from the unmilled synthetic graphite (GOS), which had concentrations of 18.6% C–OH, 9.9% C–O–C, 37.3% C=O, and 11.5% COOH. Furthermore, GOS showed 22.8% carbon structure with sp^2^ and sp^3^ hybridization, which decreased to 12.7% in GOSm because of the attrition process. This resulted in a decrease in the C/O ratio from 2.1 to 1.8. Additionally, GO obtained from milled amorphous graphite (GOAm) also increased in C–OH, C–O–C, and COOH functional groups from 29.1, 1, and 14.2% to 31.7, 6.7, and 22.7%, respectively. This is comparable to GO from amorphous graphite without attrition milling (GOA), showing a decrease of the C/O ratio from 1.2 to 0.8, thus suggesting an increase in the oxidation degree.

**Table 1:** Percent binding quantification by XPS for C1s of GO synthesized from graphite flakes (GOG), milled graphite flakes (GOGm), synthetic graphite (GOS), milled synthetic graphite (GOSm), amorphous graphite (GOA), and milled amorphous graphite (GOAm)

	sp^2^/sp^3^ (284.7 ± 0.1 eV)	C–OH (285.7 ± 0.2 eV)	C–O–C (286.7 ± 0.2 eV)	C=O (287.6 ± 0.1 eV)	COOH (289.0 ± 0.1 eV)	C/O
GOG	33.8	22.1	29.2	13.0	1.1	1.3
GOGm	42.9	8.9	8.7	26.3	13.2	1.8
GOS	22.8	18.6	9.9	37.3	11.5	2.1
GOSm	12.7	22.6	11.6	38.0	15.2	1.8
GOA	16.2	29.1	1.0	39.6	14.2	1.2
GOAm	3.2	31.7	6.7	35.7	22.7	0.8

This increase in oxygen-bearing functional groups results from the mechanical milling process that promotes the exfoliation and increase of the surface area,^28^ which also enables a better diffusion and interaction of KMnO_4_ between graphene layers.^40^ Therefore, a greater number of hydroxyl groups—which is the first functional group to be generated during the oxidation of graphite—are obtained, and as the oxidation process progresses, epoxide groups (in the basal plane) and carbonyl and carboxyl groups (on the edges of the graphene sheet) are produced.^38^

The increase in the oxidation degree of GO by the use of milled graphites might also be related to their decrease in particle size.^28^ Fig. S2(a)† shows that the GO produced from graphite flakes (GOG) was composed of particles larger than 10 μm with a tortuosity characteristic of GO as a result of the introduction of sp^3^ hybridization and oxygen-bearing functional groups.^35^ Conversely, smaller particles of 3–5 μm were observed for GO obtained from milled graphite flakes as a result of the sheer force introduced by milling.^40^ The change in the oxidation degree was also evident in the changes in the tortuosity of the laminates produced; milled GO samples contained more wrinkled sheets.^3^ This variation in particle size was also observed for the other types of graphites, as GO produced from synthetic (GOSm) and amorphous graphite (GOAm) changed from approximately 10 μm for both types of graphite to 5 and 1 μm, respectively, due to the use of attrition milling (Fig. S2†). This reduction in particle size might facilitate the interaction and diffusion of H_2_SO_4_ and KMnO_4_ between the graphite layers, which give rise to the formation of oxygenated functional groups and the subsequent formation of sp^3^ bonds.^40^

**Fig. 3:**
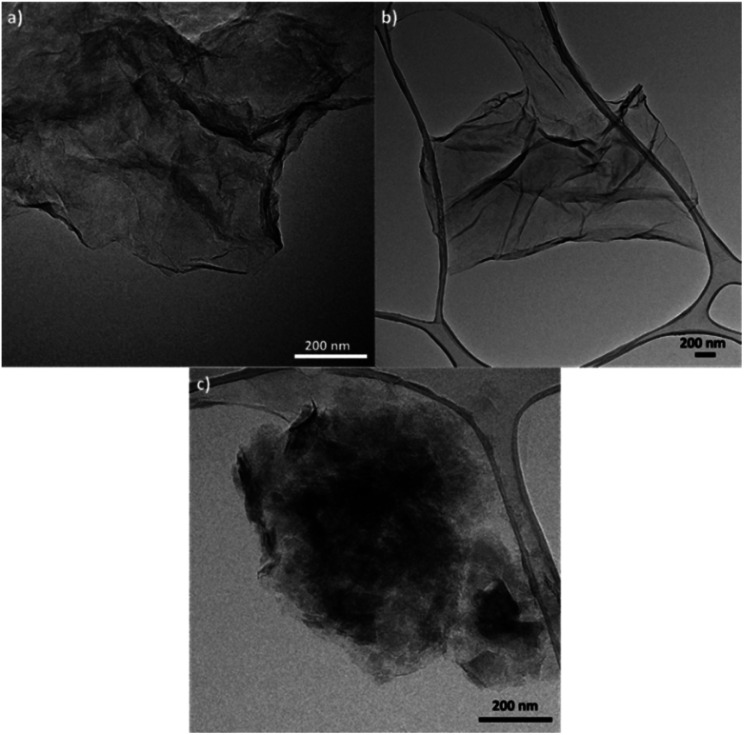
TEM images of GO synthesized from (a) milled graphite flakes (GOGm), (b) milled synthetic graphite (GOSm), and (c) milled amorphous graphite (GOAm).

TEM was also used to characterize the six types of GO obtained (Fig. 3, S3, and S4†). Fig. S3(a)† shows the microstructure of GO synthesized from graphite flakes (GOG). TEM also confirmed the formation of GOs by observing laminar structures between 0.5 and 2 μm with a distinctive tortuosity, which is characteristic of the presence of sp3 hybridization.^1,41^ Among the six samples, GO produced from milled amorphous graphite (GOAm), illustrated in Fig. 3c, showed a different microstructure compared to the rest of the GOs. This is because the large agglomerates observed appeared to be formed from smaller particles of around 50–200 nm (Fig. S4†), resulting in particles with a considerably rough microstructure compared to the laminar structure of GOSm (Fig. 3b).

**Fig. 4:**
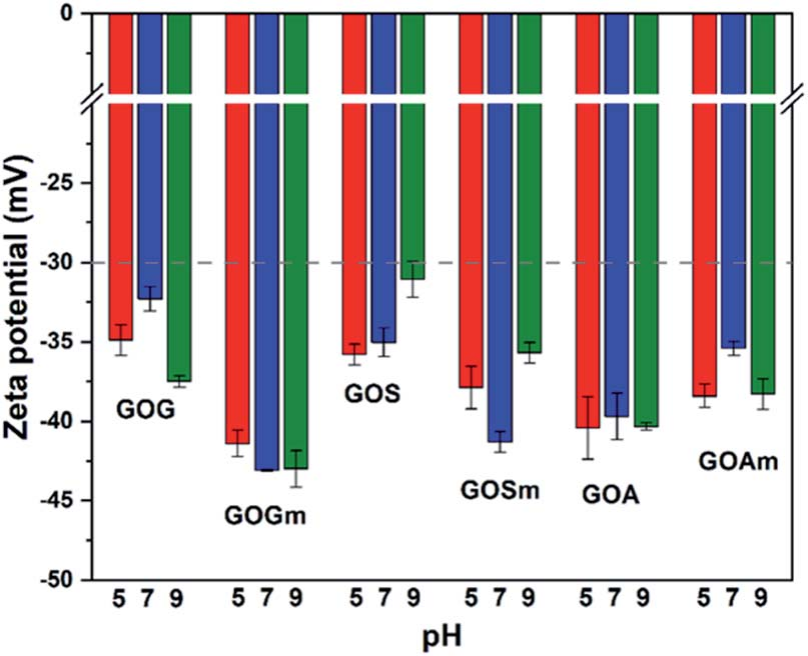
Zeta potential measurements of GO nanofluids prepared from GO synthesized from graphite flakes (GOG), milled graphite flakes (GOGm), synthetic graphite (GOS), milled synthetic graphite (GOSm), amorphous graphite (GOA), and milled amorphous graphite (GOAm). Concentration 0.02 wt%.

Fig. 4 shows the effect of pH on the ζ-potential of the various GOs. All samples had a value below −30 mV, which is the threshold of stability for a stable suspension (suspensions are considered stable above 30 mV and under −30 mV).^42,43^ GO produced from graphite flakes (GOG) had a ζ-potential between −32.3 and −37.5 mV, whereas the milled sample, GOGm, had consistently lower values of −43.1 and −42.9 mV for pHs 7–9. For GOGm, the nanofluid appeared to be more stable than GOG. This could be not only due to the reduction in particle size as described before, but also due to the large difference in –OH functional groups, since C–OH is more easily protonated compared to C–O–C and C=O.^43^ Therefore, the agglomeration of GO sheets is promoted because of the attraction between charges.^44^ On the other hand, GOG and GOGm have a higher percentage of bonds with sp^2^ and sp^3^ hybridization of all the synthesized GOs, with 33.8% for GOG and 42.9% for GOGm (Table 1). This type of structure can interact with negatively charged functional groups such as C=O and C–O–C, promoting the agglomeration of the GO sheets through its interaction with the π electrons.^3,18,23^

GO produced from synthetic graphite, GOS and GOSm, had ζ-potential values of −35.8 and −37.9 mV at pH 5 and -35.0 and −41.3 mV at pH 7, respectively. Like the previous case, the milled sample had a lower ζ-potential, suggesting higher stability in water. Conversely, the GO produced from amorphous graphite, GOA and GOAm, had a different behavior since GOA had a ζ-potential of −40.4 mV at pH 5, −39.7 mV at pH 7, and −40.3 at pH 9. Similarly, GOAm had values of −38.4 mV at pH 5, −35.4 mV at pH 7, and −38.3 mV at pH 9. For this particular sample, the use of attrition milling appeared to slightly decrease its suspension stability. This could be attributed to the fragmentation of layers by the milling process^40,45^ and the formation of fragments with a size of 50–200 nm (Fig. S4†). Compared to the relatively flat GOs produced from graphite flakes and synthetic graphite, amorphous graphite produced GO with higher roughness. This increase in the specific particle surface increases the probability of particle–particle interaction, and this variation in the shape results in a higher ζ-potential.^46^

**Fig. 5:**
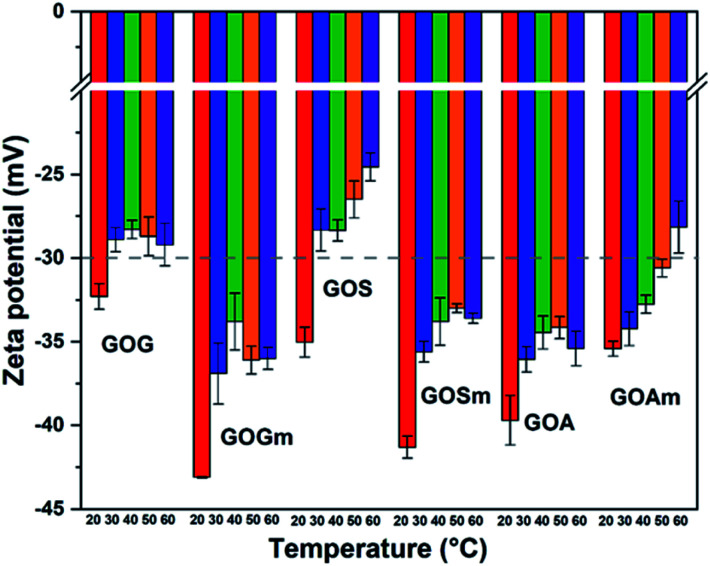
Zeta potential measurements of GO nanofluids prepared from GO synthesized from graphite flakes (GOG), milled graphite flakes (GOGm), synthetic graphite (GOS), milled synthetic graphite (GOSm), amorphous graphite (GOA), and milled amorphous graphite (GOAm). Measurements were performed at pH 7 at different temperatures, using a concentration of 0.02 wt%.

Fig. 5 shows the effect of temperature on the ζ-potential of the different GOs synthesized at pH 7. Overall, the ζ-potential increased with temperature—particularly as temperature increased from 20 to 30°C—for all the samples. For example, GOG's ζ-potential increased from −32.4 to −28.9 mV at 30 °C, while maintaining a similar value (−29.2 mV) up to 60 °C. This suggests the formation of an unstable suspension as the temperature reached 30 °C. Similarly, GOS also showed a similar reduction of stability, as its ζ-potential transitioned from −35 mV at 20 °C to −28.3 mV at 30 °C, also suggesting the formation of an unstable suspension. In general, it was observed that GO produced from milled graphite flakes and synthetic graphite produced more stable suspensions because all their values remained within the stable threshold (lower than −30 mV), despite the increase in ζ-potential at 30 °C. Conversely, unmilled GOA had higher stability compared to GOAm since its ζ-potential remained at values of −35.4 mV, even at 60 °C, compared to the milled sample, which had a clear increase in ζ-potential with temperature (−28.1 mV at 60 °C).

These variations in ζ-potential with temperature could be related to an increase in a random movement of particles in aqueous media, since temperature promotes the collision of GO layers and its consequent stacking.^47,48^ However, the values of ζ-potential also suggest that despite the temperature rise, the electrostatic repulsion between layers was strong enough to allow GOGm and GOSm to remain stable in suspension.^20^

**Fig. 6:**
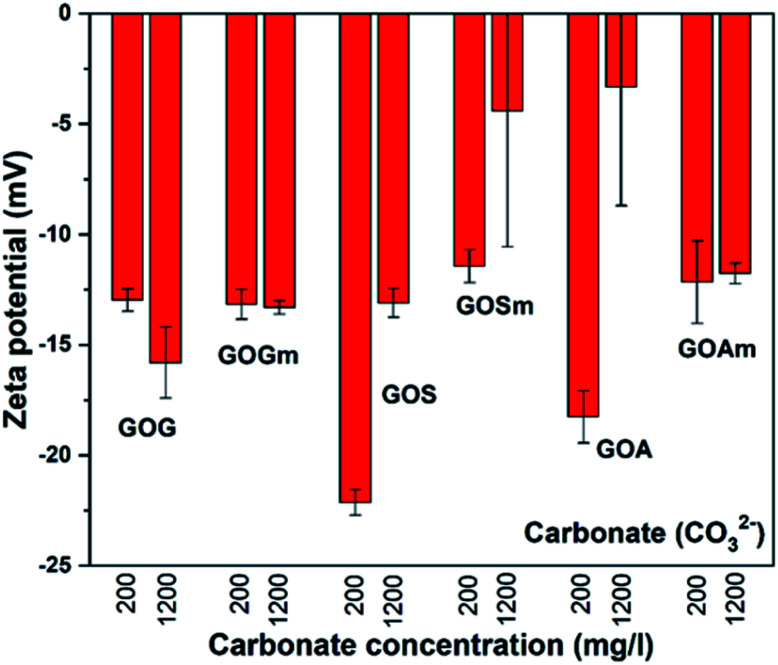
Zeta potential measurements of GO nanofluids prepared from GO synthesized from graphite flakes (GOG), milled graphite flakes (GOGm), synthetic graphite (GOS), milled synthetic graphite (GOSm), amorphous graphite (GOA), and milled amorphous graphite (GOAm) in carbonate solutions. Measurements were performed at room temperature at pH 7, using a concentration of 0.02 wt%.

As mentioned before, the water in arid and semi-arid regions can contain high concentrations of secondary salts;^24,25^ therefore, it is important to understand how these salts can affect the stability of GO. Fig. 6 shows the effect of the carbonate ion (CO_3_^2−^) on the ζ-potential of all GOs synthesized. For all GOs, ζ-potentials increased from −43.1 to −32.3 mV in deionized water to −23 and −3.4 mV in the presence of calcium carbonate, thus suggesting that all the GO suspensions became unstable and could precipitate as a result of the agglomeration of the GO sheets.^20^ For example, GOG and GOGm had very similar ζ-potential between −12.9 and −15.7 mV at 200 and 1200 mg L^−1^ CO_3_^2−^. Even GOS, with the lowest ζ-potential, reached a value of only −22.2 mV when the concentration of CO_3_^2−^ was 200 mg L^−1^. Furthermore, among all the samples, GOSm and GOA appeared to be the least stable, since at 1200 mg L^−1^ CO_3_^2−^ both had a ζ-potential in the range of −4.3 and −3.4 mV.

**Fig. 7:**
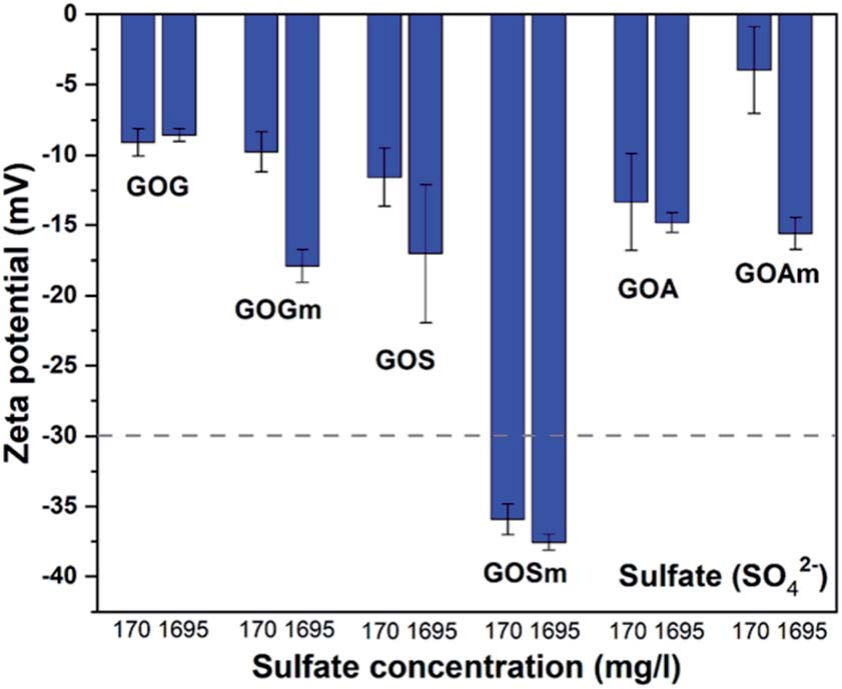
Zeta potential measurements of GO nanofluids prepared from GO synthesized from graphite flakes (GOG), milled graphite flakes (GOGm), synthetic graphite (GOS), milled synthetic graphite (GOSm), amorphous graphite (GOA), and milled amorphous graphite (GOAm) in sulfate solutions. Measurements were performed at room temperature at pH 7, using a concentration of 0.02 wt%.

Fig. 7 shows the effect of the sulfate ion (SO_4_^2−^) on the ζ-potential of GO in its lowest and highest concentrations (170 and 1695 mg L^−1^).^24,25^ It can be observed that only GO produced from milled synthetic graphite, GOSm, formed a stable suspension with ζ-potential values of −35.9 and −37.6 mV for SO_4_^2−^ concentrations of 170 mg L^−1^ and 1695 mg L^−1^, respectively. The rest of the GOs produced resulted in suspensions with higher z-potentials, ranging from −17.8 to −3.8 mV. For example, GOG and GOGm had similar values: both had a ζ-potential of −9.0 and −8.7 mV, respectively, even at the lowest concentration of 170 mg L^−1^ SO_4_^2−^.

**Fig. 8:**
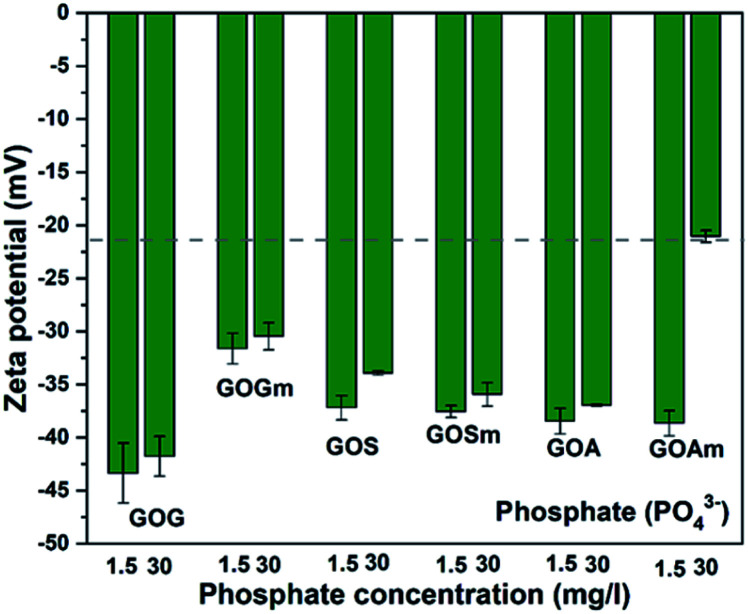
Zeta potential measurements of GO nanofluids prepared from GO synthesized from graphite flakes (GOG), milled graphite flakes (GOGm), synthetic graphite (GOS), milled synthetic graphite (GOSm), amorphous graphite (GOA), and milled amorphous graphite (GOAm) in phosphate solutions. Measurements were performed at room temperature at pH 7, using a concentration of 0.02 wt%.

Fig. 8 shows the effect of phosphate (PO_4_^3−^) on the ζ-potential of GO. In contrast to CO_3_^2−^ and SO_4_^2−^, the suspensions with PO_4_^3−^ were more stable: most of the suspensions had values below −30 mV. Overall, the ζ-potential increased with PO_4_^3−^ concentration. GOGm exhibits a lower stability at both concentrations, −31.6 mV at 1.5 mg L^−1^ and −30.5 mV at 30 mg L^−1^, compared to GOG with −43.3 at 1.5 mg L^−1^ and −41.7 at 30 mg L^−1^ mV^−1^. This difference could be related to the difference in particle size and the lower concentration of phosphate compared to other salts, since the GOG was the largest GO among all samples and smaller GO tend to agglomerate more quickly once it reaches the aggregation stage.^49^ Nevertheless, further work will be required to elucidate the origin of this higher stability. Between GOS and GOSm, there is no significant difference in terms of stability in both concentrations of PO_4_^3−^ since the ζ-potential of these GOs was between −35 and −37 mV. This could be attributed to the similarity in the content of oxygenated functional groups between these two GOs, since C–OH, C–O–C, C=O, and COOH concentrations increased from 18.6, 9.9, 37.3, and 11.5% in GOS to 22.6, 11.6, 38, and 15.2% in GOSm. On the other hand, GOA and GOAm with 1.5 mg L^−1^ PO_4_^3^- showed a similar ζ-potential of 38.6 mV; however, as the concentration increased to 30 mg L^−1^, GOAm exhibited lower stability with −21 mV. This sudden increase in ζ-potential could suggest that the surface has been saturated at this concentration due to the smallest particle size, thus promoting its agglomeration.

**Fig. 9:**
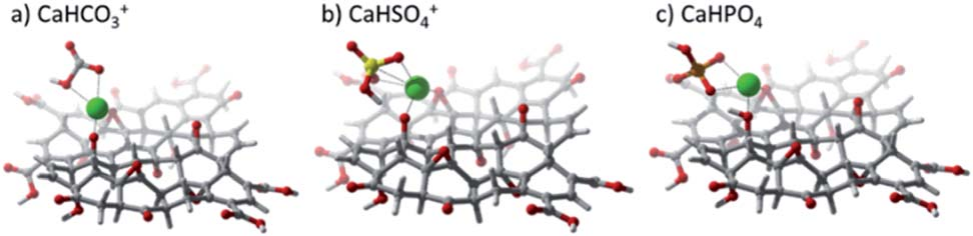
Optimized geometries calculated at the LC-ωPBE/6-31G(d,p) level of theory for calcium salts on a graphene oxide substrate. (a) CaHCO_3_^+^, (b) CaHSO_4_^+^, and (c) CaHPO_4_.

DFT calculations were used to elucidate the interaction between GO and the studied salts. Fig. 9 shows the resulting optimized structures of CaHSO_4_^+^, CaHCO_3_^+^, and CaHPO_4_, showing that the Ca^2+^ cation interacts with the GO sheet *via* hydroxyl and epoxide groups. It was also observed that anions (SO_4_^2−^ and CO_3_^2−^) were capable of removing a proton from GO, particularly from the hydroxyl group closer to the salt. Table 2 shows the interaction energies (*E*_int_) calculated. The extremely low *E*_int_ value for the CaCO_3_–GO adduct stems from the fact that the anion was very effective in withdrawing an H^+^ cation from GO. Therefore, the interaction calculated was for the CaHCO_3_^+^–GO– adduct.

**Table 2:** Interaction energies (*E*_int_) [kJ mol^−1^] calculated at the LC-ωPBE/6-31G(d,p) level of theory

Secondary salt	*E*_int_ [kJ mol^−1^]
CaHCO_3_^+^	28.30
CaHSO_4_^+^	168.94
CaHPO_4_	225.04

Our results show that water chemistry is an important factor in the stability of GO. Usually, in the aqueous phase, there are ions with a negative or positive charge that could modify the ζ-potential in different ways. For example, these ions (for example CO_3_^2−^ and Ca^2+^) could be adsorbed onto GO as a result of their interaction with the oxygenated functional groups and reduce the electrostatic repulsion or increase the attractive forces.^20,50^ These ions can also increase the hydrodynamic diameter of the particle several times by linking with the active sites on GO, thereby decreasing the electrostatic repulsion.^19^ In this case, CO_3_^2−^ and Ca^2+^ are both present in the suspension, and although all GOs still show negative surface charge, it is not enough to counter the attraction forces to stay stable in suspension. Furthermore, the binding of these ions occurs especially in those GOs that have a greater number of active sites, such as GOA and GOAm, because of their high degree of oxidation as confirmed by its C/O ratio of 1.2 and 0.8, respectively.^51^

Computer modeling suggests that the three salts might be affecting GO differently. Although CO_3_^2−^ and SO_4_^2−^ have similar concentrations, the effect of CO_3_^2−^ on the stability of GOs was more significant. This difference could be related not only to the adsorption of this ion on the surface of GO but also due to its capacity to take an H^+^ and form HCO_3_, resulting in a very low interaction energy of only 28.30 kJ mol^−1^. Previous reports have shown that carbonates (sodium and potassium carbonates) can reduce GO from 15 to 90 °C depending on pH.^52,53^ It has been suggested that GO is reduced through the formation of O_*x*_^*x*−^, possibly originating from oxygen dissolved in water or oxygen attached to the GO surface, with the production of CO_2_. Our results show that the O_*x*_^*x*−^ species could be formed on the surface of GO by the formation of a bicarbonate ion.^52^ This functional group could be available for reaction with other functional groups and promote the reduction of GO since the interaction energy of this functional group is low once the bicarbonate is formed. Our results also suggest that GO reduction by carbonates could occur without the formation of CO_2_ since the bicarbonate can be generated directly from the interaction between CO_3_ and GO.

Unlike the CO_3_^2−^ ion, the SO_4_^2−^ ion has not been reported as a reducing agent, even though compounds with sulfur content have been used for the reduction of GO.^54,55^ Although the DFT analysis showed that sulfates can also take one H^+^ from GO, the resulting CaHSO_4_^+^ still had interaction energy of 168.94 kJ mol^−1^. Hou *et al.* calculated an adsorption ratio for Ca^2+^ of 66.8% for deprotonated functional groups such as COO^−^ and 5.0 and 3.5% for groups such as COOH and –OH, respectively.^56^ On the other hand, it was also reported that SO_4_^2−^ can be attracted by the positive charge of the Ca^2+^ cation once it has been adsorbed on the surface of GO. Additionally, the hydrogen atom from the COOH group can attract the oxygen of SO_4_^2−^ with a ratio of 6.2%.^56^ Therefore, the Ca^2+^ adsorption leads to a positive surface charge, resulting in the agglomeration of GO in aqueous media.

The effect of the PO_4_^3−^ ion on the stability of GO nanofluids has not been thoroughly studied. However, its effect on the efficiency of GO as an adsorbent material has been reported to be negative since this ion could be adsorbed on GO decreasing its effectiveness.^3,57^ Zhou mentioned that phosphate interacts with the hydroxyl group by the removal of a proton.^58^ However, this behavior was not observed by the computer models in this study. Although the PO_4_^3−^ ion has the highest *E*_int_ calculated, 225.04 kJ mol^−1^, and is being adsorbed by the GO, it does not cause a significant change in the repulsive and attractive forces of the sheets, probably due to its low concentration compared to CO_3_^2−^ and SO_4_^2−^. CO_3_^2−^ has a strong impact on GO suspension stability, not only because of its strong interaction with GO but also due to its high concentrations found in desertic and semi-desertic regions.

These results demonstrate that the presence of secondary salts in the aqueous medium is a determining factor for the stability of GO nanofluids: in deionized water (Fig. 4) the ζ-potential values are lower than −30 mV for all the GO studied. However, once ions are introduced into the medium, even at low concentrations of PO_4_^3−^ (30 mg L^−1^), the stability of GOs is negatively affected. Among the salts studied, CO_3_^2−^ had the strongest impact: even at the lowest concentration relevant for semiarid regions, all the GO suspensions were unstable. Furthermore, standard GO with large particle sizes (GOG and GOS) was particularly unstable above 30°C—temperatures that can also be reached in arid and semiarid regions. These results validate that the particle size and degree of oxidation have a significant effect on GO stability in suspension but that, ultimately, secondary salts and temperature can promote their agglomeration even for small and highly oxidized structures. This could have important implications for the use of GO as a adsorbent material for metals and organic pollutants, since this material tends to agglomerate, possibly reducing its applicability for this type of application.^3,59^ Additionally, the agglomeration of GO might both promote its precipitation—thus limiting its transport in an aquatic environment—and reduce its decomposition as a result of solar/UV irradiation.^2,60^

## Conclusions

Six different GOs were synthesized from natural, synthetic, and amorphous graphite. The use of attrition milling as an initial step in the production route increased the degree of oxidation and reduced the particle size of the GO obtained. This resulted in GO with higher stability in suspension in deionized water, with ζ-potentials as low as −43.1 mV at pHs from 5–9. This suspension stability was lost by the addition of carbonate and sulfate salts at concentrations relevant to arid and semiarid regions, where carbonates can reach values as high as 1200 mg L^−1^. Carbonate had the strongest impact on GO colloidal stability since 200 mg L^−1^ considerably increased the ζ-potential up to −3.4 mV for GOSm, suggesting that GO agglomerated. The addition of calcium sulfate also negatively impacted the colloidal stability of GO, generating ζ-potentials up to −8.7 mV for a concentration of 1695 mg L^−1^. Computer models suggested that this instability in suspension could be related to the proton extraction, preferably from the hydroxyl group, resulting in a change in the surface charge and degree of oxidation of GO. To a lesser extent, temperature above 30 °C also appeared to affect GO colloidal stability, but only for large particle size GOs. These results suggest that GO will be unstable in suspension in natural water with a high concentration of carbonate and sulfate salts such as those found in arid and semi-arid regions. This phenomenon will also be exacerbated at temperatures above 30 °C in GOs obtained from natural graphite using the Marcano production route, which results in larger particle sizes. This agglomeration could limit the transport and decomposition of GO in aquatic environments.

## Author contributions

Sergio Mancillas-Salas: conceptualization, methodology, investigation, formal analysis, and writing – original draft. Ana C. Reynosa-Martinez: validation, formal analysis, writing – original draft, and writing – review and editing. Joaquin Barroso-Flores: software, validation, formal analysis, and writing – review & editing. Eddie Lopez-Honorato: conceptualization, resources, writing – review & editing, supervision, project administration, and funding acquisition.

## Conflicts of interest

There are no conflicts to declare.

## Notes

This manuscript has been authored by UT-Battelle, LLC, under contract DE-AC05-00OR22725 with the US Department of Energy (DOE). The US government retains and the publisher, by accepting the article for publication, acknowledges that the US government retains a nonexclusive, paid-up, irrevocable, worldwide license to publish or reproduce the published form of this manuscript, or allow others to do so, for US government purposes. DOE will provide public access to these results of federally sponsored research in accordance with the DOE Public Access Plan (https://energy.gov/downloads/doe-public-access-plan).

## Supplementary Material

NA-004-D2NA00070A-s001

## References

[cit1] Dreyer D. R., Park S., Bielawski C. W., Ruoff R. S. (2010). Chem. Soc. Rev..

[cit2] Gallegos-Perez W. R., Reynosa-Martinez A. C., Soto-Ortiz C., Alvarez-Lemus M. A., Barroso-Flores J., Garcia-Montalvo V., Lopez-Honorato E. (2020). Chemosphere.

[cit3] Reynosa-Martinez A. C., Navarro-Tovar G., Gallegos-Perez W. R., Rodriguez-Melendez H., Torres-Cardenas R., Mondragon-Solorzano G., Barroso-Flores J., Alvarez-Lemus M. A., Garcia-Montalvo V., Lopez-Honorato E. (2020). J. Hazard. Mater..

[cit4] Joshi R. K., Alwarappan S., Yoshimura M., Sahajwalla V., Nishina Y. (2015). Appl. Mater. Today.

[cit5] Romanchuk A. Y., Slesarev A. S., Kalmykov S. N., Kosynkin D. V., Tour J. M. (2013). Phys. Chem. Chem. Phys..

[cit6] Kuzenkova A. S., Romanchuk A. Y., Trigub A. L., Maslakov K. I., Egorov A. V., Amidani L., Kittrell C., Kvashinina O. K., Tour J. M., Talyzin A. V., Kalmykov S. N. (2020). Carbon.

[cit7] Ren X. L., Chen C., Gao Y., Chen D., Su M., Alsaedi A., Hayat T. (2018). Environ. Sci.: Nano.

[cit8] Akhavan O., Ghaderi E. (2010). ACS Nano.

[cit9] Zhao J., Wang Z., White J. C., Xing B. (2014). Environ. Sci. Technol..

[cit10] Hou W. C., Chowdhury I., Goodwin D. G., Henderson W. M., Fairbrother D. H., Bouchard D. (2015). Environ. Sci. Technol..

[cit11] Hu X., Kang J., Lu K., Zhou R., Mu L., Zhou Q. (2014). Sci. Rep..

[cit12] Thangavel S., Venogopal G. (2014). Powder Technol..

[cit13] Savazzi F., Risplendi F., Mallia G., Harrison N. M., Cicero G. (2018). J. Phys. Chem. Lett..

[cit14] Pareek S., Jain D., Shrivastava R., Dam S., Hussain S., Behera D. (2019). Mater. Res. Express.

[cit15] Hanifah M. F. R., Jaafar J., Othman M. H. D., Ismail A. F., Rahman M. A., Yusof N., Salleh W. N. W., Aziz F. (2019). Materialia.

[cit16] Meng X., Bang S., Korfiatis G. P. (2000). Water Res..

[cit17] Su C., Puls R. W. (2001). Environ. Sci. Technol..

[cit18] Chowdhury I., Duch M. C., Mansukhani N. D., Hersam M. C., Bouchard D. (2013). Environ. Sci. Technol..

[cit19] Lanphere J. D., Rogers B., Luth C., Bolster C. H. (2014). Environ. Eng. Sci..

[cit20] Gudarzi M. M. (2016). Langmiur.

[cit21] Wang M., Gao B., Tang D., Sun H., Yin X., Yu C. (2018). Colloids Surf., A.

[cit22] Hua Z., Tang Z., Bai X., Zhang J., Yu L., Cheng H. (2015). Environ. Pollut..

[cit23] Chowdhury I., Mansukhani N. D., Guiney L. M., Hersam M. C., Bouchard D. (2015). Environ. Sci. Technol..

[cit24] Fernandez-Luqueno F. (2016). Int. J. Environ. Pollut..

[cit25] Navarro-Noya Y. E., Suarez-Arriaga M. C., Rojas-Valdes A., Montoya-Ciriaco N. M., Gomez-Acata S., Fernandez-Luqueno F., Dendooven L. (2013). Microb. Ecol..

[cit26] Carbajal-Martinez D., Peiffer L., Hinojosa-Corona A., Trasvina-Castro A., Arregui-Ojeda S. M., Carranza-Chavez F. J., Flores-Luna C., Mendez-Alonso R., Inguaggiato C., Casallas-Moreno K. L. (2021). Renewable Energy.

[cit27] Marcano D. C., Kosynkin D. V., Berlin J. M., Sinitskii A., Sun Z., Slesarev A., Lawerence B. A., Wei L., Tour J. M. (2010). ACS Nano.

[cit28] Mancillas-Salas S., Barroso-Flores J., Villaurrutia J., Garcia-Montalvo V., Lopez-Honorato E. (2020). Ceram. Int..

[cit29] Fairley N. , Introduction to XPS and AES, Casa XPS Manual, 2009

[cit30] Ganguly A., Sharma S., Papakonstantinou P., Hamilton J. (2011). J. Phys. Chem. C.

[cit31] Desimoni E., Casella G. I., Morone A., Salvi A. M. (1990). Surf. Interface Anal..

[cit32] Frisch M. J. , Trucks G. W., Schlegel H. B., Scurseria G. E., Robb M. A. and Cheeseman J. R., Gaussian 09, Gausssian, Inc., Wallingford CT, 2016

[cit33] Huang L., Zhang M., Li C., Shi G. (2015). J. Phys. Chem. Lett..

[cit34] Glendening E. D. , Reed A. E., Carpenter J. E. and Weinhold F., NBIO Version 3.1, Wisconsin, 1996

[cit35] Xu S., Liu J., Xue Y., Wu T., Zhang J. (2017). Fullerenes, Nanotubes, Carbon Nanostruct..

[cit36] Li Z. Q., Lu C. J., Xia Z. P., Zhou Y., Luo Z. (2007). Carbon.

[cit37] Szabo T., Berkesi O., Forgo P., Josepovits K., Sanakis Y., Petridis D., Kekany I. (2006). Chem. Mater..

[cit38] Liu Z., Duan X., Zhou X., Qian G., Zhaou J., Yuan W. (2014). Ind. Eng. Chem. Res..

[cit39] Koono H. , in Materials Science and Engineering of Carbon: Characterization, ed. H. Konno, Elsevier, Pekin, 2016, pp. 153–171

[cit40] Shojaeenezhad S. S., Farbod M., Kazeminezhad I. (2017). J. Sci.: Adv. Mater. Devices.

[cit41] Stobinski L., Lesiak B., Malolepszy A., Mazurkiewicz M., Mierzwa B., Zemek J., Jiricek P., Bieloshapka I. (2014). J. Electron Spectrosc. Relat. Phenom..

[cit42] ASTM, ASTM D 4187-82 . Zeta Potential of Colloids in Water and Wastewater, American Society for Testing and Materials, 1985

[cit43] Konkena B., Vasudevan S. (2012). J. Phys. Chem. Lett..

[cit44] Krishnamoorthy K., Veerapandian M., Yuri K., Kim S. J. (2013). Carbon.

[cit45] Fan T., Zeng W., Tang W., Yuan C., Tong S., Cai K., Liu Y., Huang W., Min Y., Epstein A. J. (2015). Nanoscale Res. Lett..

[cit46] Au P. I., Leong Y. K., Liu J. (2014). Colloids Surf., A.

[cit47] Yu W., Xie H., Chen W. (2014). J. Appl. Phys..

[cit48] Hajjar Z., Rashidi A. M., Gohozatloo A. (2014). Int. Commun. Heat Mass Transfer.

[cit49] Tang h., Zhao Y., Yang X., Liu D., Shao P., Zhu Z., Shan S., Cui F., Xing B. (2017). Environ. Sci. Technol..

[cit50] Khosrojerdi S., Lavasani A. M., Vakii M. (2017). Sol. Energy Mater. Sol. Cells.

[cit51] Navalon S., Dhakshinamoorthy A., Alvaro M., Antonietti M. (2017). Chem. Soc. Rev..

[cit52] He D., Peng Z., Gong W., Luo Y., Zhao P., Kong L. (2015). RSC Adv..

[cit53] Jin Y., Huang S., Zhang M., Jia M., Hu D. (2013). Appl. Surf. Sci..

[cit54] Chen W., Yan L., Bangal P. R. (2010). J. Phys. Chem. C.

[cit55] Reynosa-Martinez A. C., Gomez-Chayres E., Villaurrutia R., Lopez-Honorato E. (2021). Materials.

[cit56] Hou D., Zhang Q., Wnag M., Zhang J., Wang P., Ge Y. (2019). Comput. Mater. Sci..

[cit57] Wan W., Pepping T. J., Banerji T., Chaudhari S., Giammar D. E. (2011). Water Res..

[cit58] Zhou G., Jin B., Wang Y., Dong Q., Maity A., Chang J., Ren R., Pu H., Sui X., Mao S., Chen J. (2020). Mol. Syst. Des. Eng..

[cit59] Nupearachchi C. N., Mahatantila K., Vithanage M. (2017). Groundw. Sustain. Dev..

[cit60] Zhao Y., Liu Y., Zhang X., Liao W. (2021). Chemosphere.

