# Supplementary material for: Impact of secondary salts, temperature, and pH on the colloidal stability of graphene oxide in water
Source: Nanoscale Adv. 2022 Apr 22;4(11):2435–43. doi: 10.1039/d2na00070a (PMC9418902; doi:10.1039/d2na00070a)
Supplement: NA-004-D2NA00070A-s001 [file NA-004-D2NA00070A-s001.pdf]

Electronic Supplementary Material (ESI) for Nanoscale Advances.
This journal is © The Royal Society of Chemistry 2022

**Impact of secondary salts, temperature, and pH on the colloidal stability of graphene oxide in water**

S. Mancillas-Salas[a], A. C. Reynosa-Martínez[a], J. Barroso-Flores[b,c], E. López-Honorato[a,d1]

[a] Centro de Investigación y de Estudios Avanzados del IPN (CINVESTAV), Unidad Saltillo, AV. Industria Metalúrgica 1062, Ramos Arizpe, 25900, México

[b] Instituto de Química, Universidad Nacional Autónoma de México, Circuito Exterior, Ciudad Universitaria, México, 04510, D.F., Mexico.

[c] Centro Conjunto de Investigación en Química Sustentable UAEM-UNAM, Carretera Toluca-Atlacomulco Km 14.5, Unidad San Cayetano, Toluca, Estado de México, 50200 México.

[d] Oak Ridge National Laboratory, Oak Ridge, TN 37831, United States

[1] Corresponding author. E-mail: honoratole@ornl.gov
These authors contributed equally: S. Mancillas-Salas and A. C. Reynosa-Martínez.

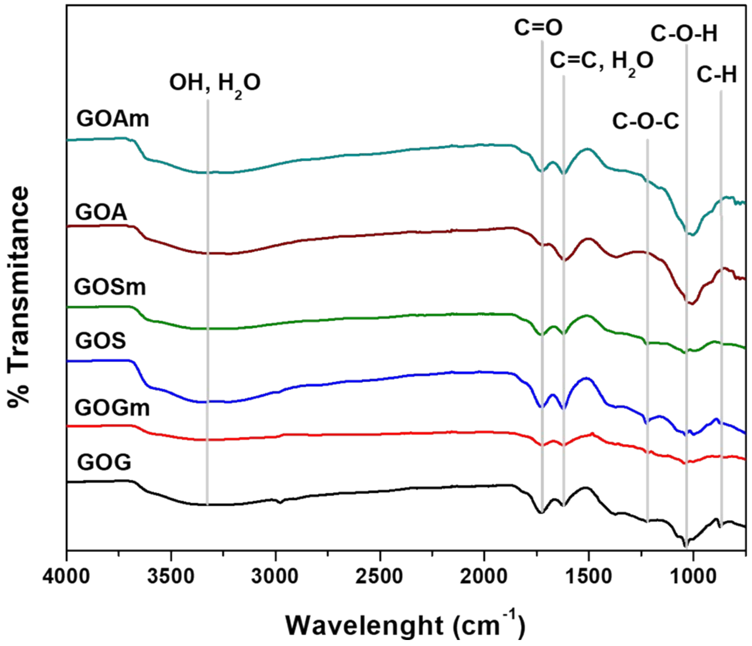


Figure S1. FTIR spectra of GO synthesized from graphite flakes (GOG), milled graphite flakes (GOGm), synthetic graphite (GOS), milled synthetic graphite (GOSm), amorphous graphite (GOA), and milled amorphous graphite (GOAm).

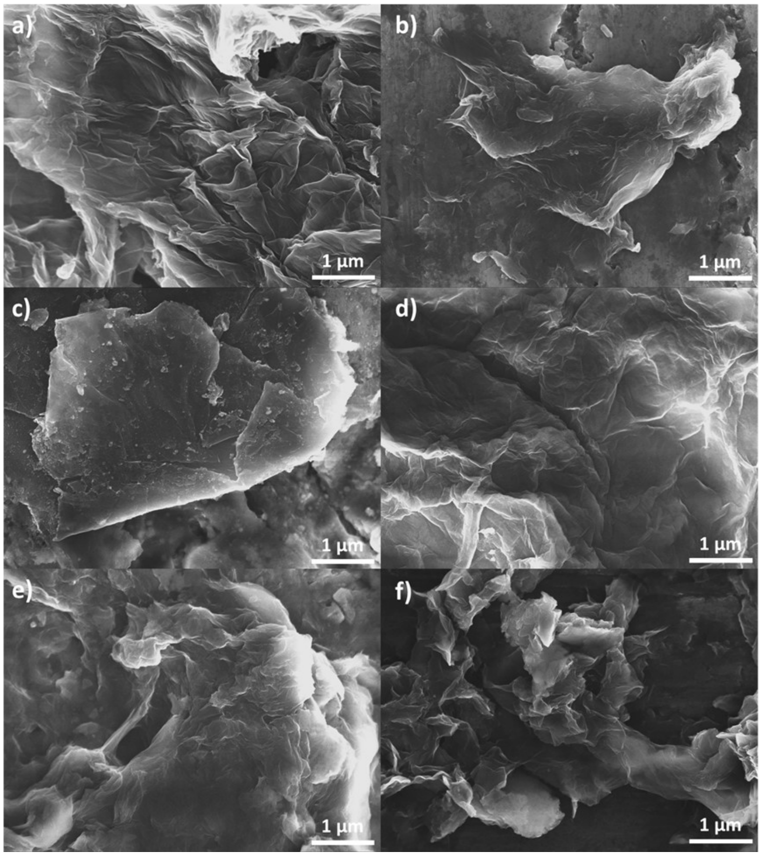


Figure S2. FE-SEM images of GO synthesized from a) graphite flakes (GOG), b) milled graphite flakes (GOGm), c) synthetic graphite (GOS), d) milled synthetic graphite (GOSm), e) amorphous graphite (GOA), and f) milled amorphous graphite (GOAm).

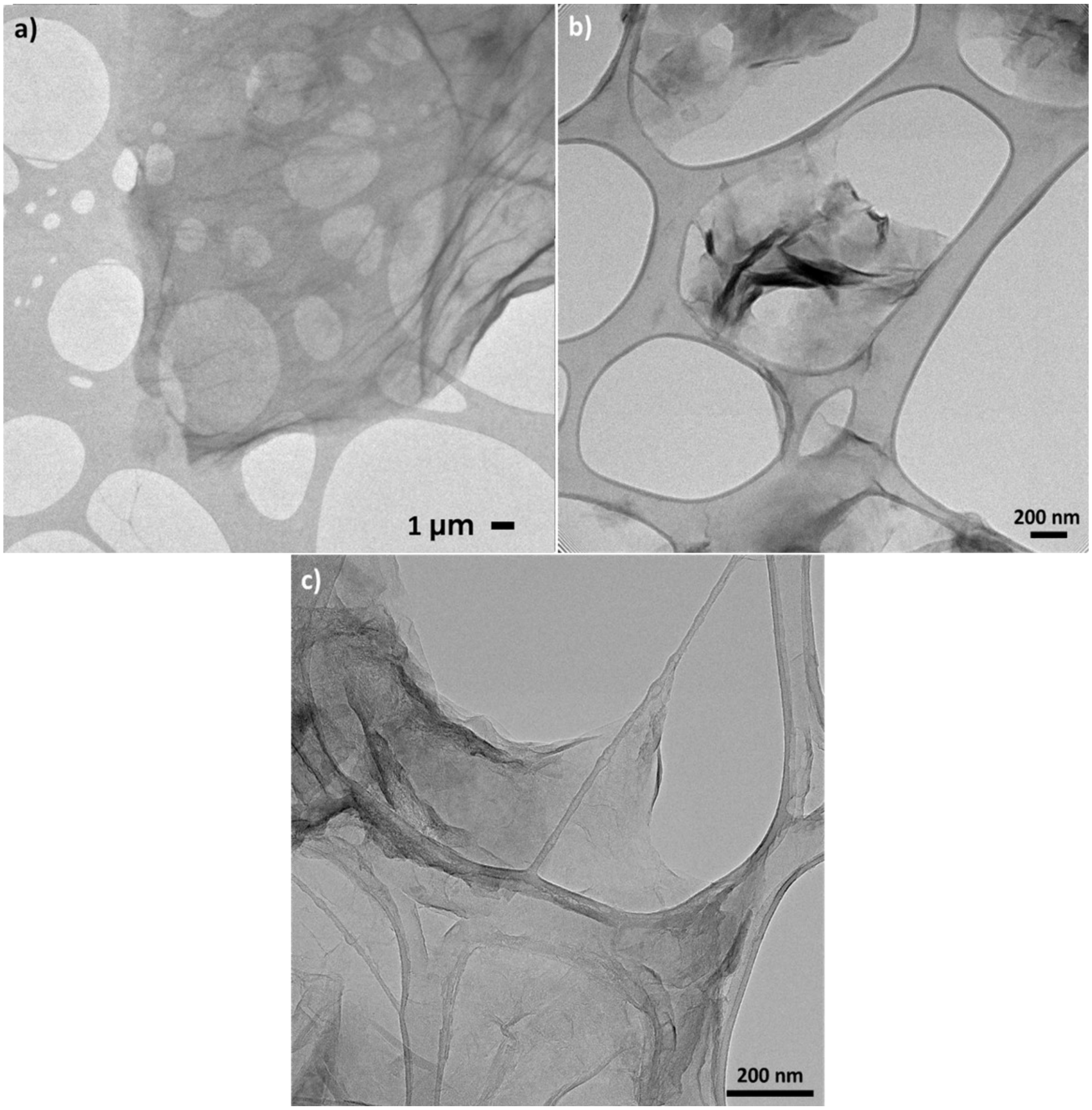


Figure S3. TEM images of GO synthesized from a) graphite flakes (GOG), b) synthetic graphite (GOS) and c) milled amorphous graphite (GOA).

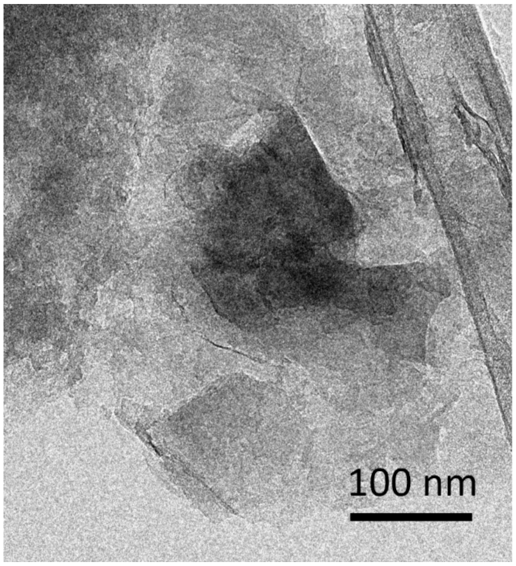

Figure S4. TEM image of GO synthetized from milled amorphous graphite.
